# The Army Surgeons

**Published:** 1886-02

**Authors:** Frank H. Hamilton

**Affiliations:** New York, 43 W. 32d St.


### THE ARMY SURGEONS.

NEW YORK, 43 W. 32d St., January 1, 1886.

*Editors Buffalo Medical and Surgical Journal:*

The enclosed manuscript was written, as you will see, on the "field" in 1862, twenty-three years ago. As it would have been manifestly improper to have published it while I remained in the army, I sent it to Mrs. Hamilton, with instructions to keep it until I authorized its publication. It has remained in her charge ever since, and has only been brought to my notice recently, and since her decease.

It now seems to me, although the provocations under which it was written have long been forgotten, that it might be proper to publish it, in view of the facts that the criticisms remain in print and may be recalled by some one, and that no one has, so far as I know, taken the pains to answer them. I am influenced now to offer them to you, because there is just now a period of revival of reminiscences of the war, but in which only officers of the line appear conspicuously as men who rendered to their country great public service.

HARRISON'S LANDING, Va., July 30, 1862.
GEN'L KEYES' HEADQUARTERS.

We have listened for some time patiently to the censures laid upon the medical corps of the army, trusting that time and occasion would furnish a vindication. The censures to which we refer have seldom a direct personal application, but are usually in general terms, implicating all, or nearly all, who are now serving in the various portions of the U. S. Army, whether regular, volunteer or contract surgeons.

The writer proposes to reply briefly, but as specifically as the general nature of the charges and criticisms will permit.

The medical officers attached to the *regular* army have all been subjected to an examination as to their moral, physical and professional qualifications prior to admission, notoriously severe, and in all respects equal to that of those officers who have been graduated at West Point or at Annapolis. In no country in Europe is the test more rigid. In England it is much less rigid.

*Volunteer surgeons*, or surgeons attached to the volunteer army, have been subjected to similar tests, differing more or less in standard in the different States of the Union; but in no case which has come to our knowledge, have these examinations been omitted.

*Contract surgeons* have all been examined by an army medical board, as have also *Brigade surgeons;* the board in the latter case being composed of some of the ablest regular army officers.

At first, and for many months after the war began, no officer of the volunteer service *not* of the medical profession was, in a single instance, subjected to an examination of any kind, nor, in general, were any testimonials required as to intellectual or moral qualifications.

These facts alone must establish a presumption that the medical officers of the army are as well qualified for the performance of their duties as the officers of the other departments; but there is one other fact, not yet stated, and which is entitled to consideration. The duties to which surgeons are called, are not new to them. There is nothing in the experience of a camp, except the mere routine of business transactions, to which they have not been previously trained and accustomed. The diplomas alone, conferring the degree of doctor in medicine, might be justly regarded as testimony on this point; but, in addition to this, a very large proportion of these gentlemen have been several years engaged in active private practice, or have been in charge of hospitals. Most of the brigade surgeons were taken from the highest ranks of the profession, and are men whose social position at home was eminently respectable.

The presumption, therefore, is established in our favor, and we might properly decline any further argument until the charges are more specifically made; but, from respect to the public, who, remote from the scene of our labors, look alone to the press for a confirmation or denial of these criminations, we shall enter upon our own defense.

Where have surgeons failed of their duty? On the field of battle? We have been on the field, or near enough to observe the conduct of the surgeons attached to the various regiments engaged, sufficiently often to say that in this respect they have seldom done less than their duty. We have seen them under fire, deliberately dressing the wounds of soldiers, administering to them nourishment, stimulants and water. We have seen them going forward with the litter-bearers to the very field of conflict, and assisting the men to carry off the wounded.

In many instances during the present war, surgeons, in the performance of these and similar duties, have been wounded, and some have died from the wounds thus received.

If others have met with examples of surgeons who have deserted their posts at such a time, it has not occurred to us, after a pretty long experience, to see them; while, in the same experience, it has occurred to us to see scores of men and officers, belonging to the *fighting* portion of the army, file past the depots which we had established to the rear, and whom, since they were not wounded, we had no occasion to detain in places not yet beyond the reach of bullets.

Have surgeons neglected their duties on the field after the battle has closed? Not to our knowledge. On the contrary, they have labored night and day, for one, two and three successive days, without sleep, without rest, and sometimes almost without food, until, from sheer exhaustion, they have been compelled to desist. After a battle, the surgeon is usually left alone to do his work. His nurses, cooks and servants are gone. His wounded are in deserted houses, in barns or upon the open field. He has often no lights, no seats, no beds, no tables, no cooking utensils, no food or stimulants. Everything almost has to be improvised. No commissary has ever been known, of his own instance, to bring supplies of food, or quartermaster, of his own instance, to furnish conveyance. We have to this day never found at hand a commissary or quartermaster immediately before, immediately after, or during a battle. We have repeatedly been obliged to leave our appropriate work and go a long distance in pursuit of a commissary, and, when found, we have been obliged to return without the food necessary to save our men from famine, because no transport could be obtained to convey the provisions.

In long and forced marches, both in the advance and in the retreat, surgeons have done constantly all that lay in their power to help along the disabled, and to provide for those who must be left. It is no part of a surgeon's duty to transport the sick. This duty, by regulation, belongs to the quartermaster, but in a very large majority of cases, where troops are suddenly ordered to move, surgeons find it necessary to attend to this themselves; but the means of transportation are seldom equal to the demand. The army regulations declare that the following schedule of transports for the sick and wounded, and for hospital supplies, will be adopted for a state of war with a civilized enemy: "For a regiment of ten companies, two four-wheeled ambulances, ten two-wheeled ambulances and four two-wheeled transport carts;" or transportation for about forty men to each regiment, it being estimated that about this proportion of men will be too ill to march; while the fact is, that in that portion of the army to which we are at present attached, there is not, and there has not been for a long time, one ambulance to a regiment; or, to state it in round numbers, where transportation ought to have been supplied for 2,000 men, there has been actually transportation for only 200.

Transportation carts for hospital stores, hospital tents, etc., have been supplied in the proportion of not more than one to two regiments.

It is a common practice, moreover, to turn over to the hospital department, for the ambulances and transports, the poorest horses, and in some instances, regimental, brigade and division quartermasters, having received with the ambulances good horses, have selected the best and exchanged them with other departments of the service for inferior horses; so that one seldom sees before an ambulance sound horses, unless the horses have been furnished by the State from which the regiment came. From all these facts, and not from any fault or negligence of the surgeon, it results, that hospital tents, with blankets, cots, medicines, and other hospital supplies, are often left behind; some of which have been finally recovered, but most of which have been utterly lost, at least, to the regiments to which they belonged. Ambulances sent forward to recover the wounded and bring them to the rear, are abandoned in the road, the horses having balked, or becoming stalled in the mud: sick, wounded and weary men straggle and are left by the roadside.

On the day following a heavy-forced march, the surgeon is notified that sick men, belonging to the command, are reported to have been left several miles in the rear without food or water. The company officers, whose duty it is to prevent straggling, have permitted these men one by one to fall out, for the good reason that it was found impossible for them to keep up.

The surgeon, or his assistant, must, in addition to the performance of his other duties, ride back ten or fifteen miles, and provide for them as well as he can; and it is not improbable that instead of finding only one squad of such disabled men, he will find three or four at different points of the road who have crawled under some old shed, or other similar shelter, sick, exhausted, without blankets or food, and utterly helpless.

The health of the troops has always an intimate relation with the sanitary police of the camp. No one understands this better than a medical man, for the relations between health, cleanliness, and purity of air, constitute a large portion of his study and education; but it is not in the power of any regimental surgeon to enforce camp police without the approval and cooperation of the commanding officers, especially of the colonel. We do not think any person can fully appreciate the difficulty under which the surgeon labors in this respect, until he has had personal experience. He has no authority to command, except as in some instances the commanding officer invests him with such authority. He can only suggest; but he may suggest and report until doomsday, yet, unless the colonel issues the order, not a street will be cleaned, not a tent will be struck, not a trench or sink will be dug. We have seen encampments in a most perfect condition of police, where the surgeons have acknowledged to us that they never in any way interfered with it—the colonels had themselves established their rules, and their company officers had, by daily inspections and personal attentions, carried out the letter and spirit of the orders—while in other instances, and these examples we are bound to say are the most frequent, we have seen encampments badly policed, where intelligent surgeons, of neat and systematic habits, have labored zealously for many months, but whose efforts were rendered completely ineffective from the indifference and want of support on the part of the colonel.

Field surgery has been made the subject of unfriendly criticism by those who have been permitted to see our patients after the lapse of several days; yet intelligent medical men never, or very seldom, venture to judge of the propriety of an operation, or of the skill exercised in its execution by the condition of the patient, or of his wound at this period. The best-made stumps may ulcerate, slough and open, the skin and muscles may retract and expose the bones from faults of the constitution, from the severity of the shock attending the accident, from exposure to heat or cold, from rude jolting over rough roads, or from rude handling on the part of those who move them from place to place.

The neatest dressings may become disarranged and soiled; and in hot climates, where wounds cannot be sufficiently protected, magots will obtrude their disgusting presence in spite of the utmost diligence of the surgeon and his attendants.

If, however, it could be clearly shown that there was a good deal of bad surgery in army practice—that operations are not always judiciously timed, or skillfully made, or the wounds neatly dressed; does it justify the severity of the judgment sometimes given in these cases? Have any of these gentlemen who find it so easy, and who feel it so much their duty, to point out our errors ever made an amputation "under fire?" It is no uncommon thing for a surgeon on the field to be compelled to change his position once or twice during an operation, on account of a change in the direction or range of the shots.

Such interruptions, together with the urgency of the claims of other wounded men lying about, render it necessary to decide quickly, and, no doubt, sometimes injudiciously, and to execute rapidly.

In short, while we are no apologists for the surgeon who neglects his duties, or performs them unskillfully through ignorance, or who, from any cause or in any degree, fails of doing all that can reasonably be demanded of him, we protest against ill-grounded, uncharitable and wholesale criminations of a corps of officers who have thus far in this war performed their duties, we believe, as faithfully and as skillfully as any other corps in the army, yet for whom there are but few of those incentives to good conduct which are placed before other officers of the line and staff. A surgeon may be commended by his commanding officer for diligence and bravery in the discharge of his appropriate duties, and occasionally this has been done; yet not, we think, as often as the commendation has been merited; but in no case has there been placed before him the incentive of promotion. In the medical department of the U. S. Army, there is provision for one brigadier-general, one colonel, and eight lieutenant-colonels. Only ten medical men can ever rise above the rank of major, while, in the other departments of the service, provision is made for over 2,000 who shall rank above major. Surgeons may be called to offices of additional trust and responsibility, involving usually additional expense, but their rank and pay must remain the same.

To the above, I wish to add a few supplementary remarks:

According to the report of the surgeon-general, (Medical and Surgical History of the War of the Rebellion, 1861–65, Surgical Volume, Part I., pages 30–32 of Appendix,) including the period from the beginning of the war to 1865, 28 medical officers were killed, or died of wounds received in action, thirteen were killed by partisan troops, guerrillas, etc., nine died of wounds received in the line of duty, and seventy-three were wounded in action, but recovered.

This report was made up in 1865, or so as to include the year 1865, but it was not published until 1870, and it is probable, therefore, that the pension rolls, with other and later sources of information, would considerably increase the number of those who died of fatal injuries.

I have not been able to find in the surgeon-general's reports a statement as to how many medical officers died of disease contracted in service, but I have in my possession a memorandum, dated December 6, 1869, to the effect that Dr. Woodward gave the number of those medical officers who died of disease as 385, including four who died in prison.

From what I have seen in the medical journals and elsewhere, I presume an equal number of medical men were killed and died of diseases in the Confederate Army.

Yours truly,

FRANK H. HAMILTON.

